# Microaerobic Lifestyle at Nanomolar O_2_ Concentrations Mediated by Low-Affinity Terminal Oxidases in Abundant Soil Bacteria

**DOI:** 10.1128/mSystems.00250-21

**Published:** 2021-07-06

**Authors:** Daniela Trojan, Emilio Garcia-Robledo, Dimitri V. Meier, Bela Hausmann, Niels Peter Revsbech, Stephanie A. Eichorst, Dagmar Woebken

**Affiliations:** a Division of Microbial Ecology, Department of Microbiology and Ecosystem Science, Centre for Microbiology and Environmental Systems Science, University of Vienna, Vienna, Austria; b Department of Biology, Faculty of Marine and Environmental Sciences, University of Cádiz, Cádiz, Spain; c Joint Microbiome Facility of the Medical University of Vienna and the University of Vienna, Vienna, Austria; d Department of Laboratory Medicine, Medical University of Vienna, Vienna, Austria; e WATEC, Department of Biology, Aarhus University, Aarhus, Denmark; UiT—The Arctic University of Norway

**Keywords:** terminal oxidase, oxygen, acidobacteria, kinetics, transcriptomics

## Abstract

High-affinity terminal oxidases (TOs) are believed to permit microbial respiration at low oxygen (O_2_) levels. Genes encoding such oxidases are widespread, and their existence in microbial genomes is taken as an indicator for microaerobic respiration. We combined respiratory kinetics determined via highly sensitive optical trace O_2_ sensors, genomics, and transcriptomics to test the hypothesis that high-affinity TOs are a prerequisite to respire micro- and nanooxic concentrations of O_2_ in environmentally relevant model soil organisms: acidobacteria. Members of the *Acidobacteria* harbor branched respiratory chains terminating in low-affinity (*caa*_3_-type cytochrome *c* oxidases) as well as high-affinity (*cbb*_3_-type cytochrome *c* oxidases and/or *bd*-type quinol oxidases) TOs, potentially enabling them to cope with varying O_2_ concentrations. The measured apparent *K_m_* (*K_m_*_(app)_) values for O_2_ of selected strains ranged from 37 to 288 nmol O_2_ liter^−1^, comparable to values previously assigned to low-affinity TOs. Surprisingly, we could not detect the expression of the conventional high-affinity TO (*cbb*_3_ type) at micro- and nanomolar O_2_ concentrations but detected the expression of low-affinity TOs. To the best of our knowledge, this is the first observation of microaerobic respiration imparted by low-affinity TOs at O_2_ concentrations as low as 1 nM. This challenges the standing hypothesis that a microaerobic lifestyle is exclusively imparted by the presence of high-affinity TOs. As low-affinity TOs are more efficient at generating ATP than high-affinity TOs, their utilization could provide a great benefit, even at low-nanomolar O_2_ levels. Our findings highlight energy conservation strategies that could promote the success of *Acidobacteria* in soil but might also be important for as-yet-unrevealed microorganisms.

**IMPORTANCE** Low-oxygen habitats are widely distributed on Earth, ranging from the human intestine to soils. Microorganisms are assumed to have the capacity to respire low O_2_ concentrations via high-affinity terminal oxidases. By utilizing strains of a ubiquitous and abundant group of soil bacteria, the *Acidobacteria*, and combining respiration kinetics, genomics, and transcriptomics, we provide evidence that these microorganisms use the energetically more efficient low-affinity terminal oxidases to respire low-nanomolar O_2_ concentrations. This questions the standing hypothesis that the ability to respire traces of O_2_ stems solely from the activity of high-affinity terminal oxidases. We propose that this energetically efficient strategy extends into other, so-far-unrevealed microbial clades. Our findings also demonstrate that physiological predictions regarding the utilization of different O_2_ concentrations based solely on the presence or absence of terminal oxidases in bacterial genomes can be misleading.

## INTRODUCTION

Oxygen (O_2_) has a high redox potential (E_0_′ = +0.82 V), which, together with its ubiquity, makes it a favorable electron acceptor for energy generation. The concentration of O_2_ across numerous microbial habitats can vary from saturation to anoxia (1). It is believed that aerobic microorganisms meet these fluctuating conditions by harboring low- and high-affinity terminal oxidases (TOs), presumably allowing them to use a wide range of O_2_ concentrations.

Terminal oxidases, which mediate the final redox reaction in the electron transport chain (ETC) during aerobic respiration, are grouped into three superfamilies: (i) heme-copper oxidases (HCOs), (ii) cytochrome *bd*-type oxidases, and (iii) alternative oxidases. HCOs are multisubunit complexes and function as cytochrome *c* or as quinol oxidases, contributing to energy conservation, the generation of a proton motive force, O_2_ scavenging, and maintaining redox homeostasis (2, 3). Based on overall amino acid similarities of the catalytic subunits and differences of the proton channels, the HCO superfamily is classified into three families: A (subfamilies A1 and A2), B, and C (4). Family A oxidases have a low affinity for O_2_, with a reported Michaelis-Menten constant (*K_m_*) for O_2_ of 200 nmol O_2_ liter^−1^ (5). HCO families B and C are considered high-affinity TOs with high catalytic activity at low O_2_ concentrations but reduced proton-pumping efficiency (6), with *K_m_* values for the family C *cbb*_3_-type oxidases of 7 to 40 nmol O_2_ liter^−1^ (7–9). The high-affinity cytochrome *bd*-type oxidase encoded by the *cydAB* genes (10–12) has reported *K_m_* values of 3 to 8 nmol O_2_ liter^−1^ (13). Cytochrome *bd*-type oxidases do not pump protons across the membrane but contribute to proton motive force by using electrons from the extracytoplasmic side and protons from the cytoplasmic side (11).

High-affinity TOs are believed to sustain energy conservation at diminishing concentrations by enabling respiration at trace amounts of O_2_ (i.e., micromolar O_2_ concentrations) (14–16). Although there has been some suggestion that low-affinity TOs are present at micromolar O_2_ concentrations in addition to high-affinity TOs (5), it remains unclear if the low-affinity TOs can actively and even solely contribute to respiration at these O_2_ concentrations. At nanomolar O_2_ concentrations, microorganisms transition from aerobic respiration to anaerobic-based metabolism (substrate-level phosphorylation or anaerobic respiration), referred to as the Pasteur point (17, 18). To the best of our knowledge, gene expression-based investigations of terminal oxidases at nanomolar O_2_ concentrations are scarce (e.g., Gong et al. reported expression at O_2_ levels of ≤200 nmol [19]), and therefore, it is mostly speculated that the high-affinity terminal oxidases are primarily responsible for energy production at low-nanomolar O_2_ concentrations.

In soil, O_2_ availability can be spatially and temporally dynamic, depending on the edaphic properties and microbial activity (20, 21). As such, microbial survival in soil is dependent on the ability to adapt to changes in local O_2_ conditions. Environmental data and genome surveys suggest that both low- and high-affinity TOs are widely distributed in soils (16). *Acidobacteria* represent one of the most abundant and phylogenetically diverse phyla in soils worldwide (22–24) and are assigned a central role in carbon mineralization and plant polymeric carbon degradation (25, 26). Genes encoding high- and low-affinity TOs have been identified in several genomes of the phylum *Acidobacteria* (27), suggesting the capacity to respire across a wide gradient of O_2_ concentrations. As respiratory flexibility can be attained through branched respiratory chains that terminate in multiple oxidases with different affinities for O_2_ (15), this facet might be key to their ecological success in soil.

Using *Acidobacteria* as model soil organisms, we explored respiratory kinetics and evaluated their gene expression using whole-transcriptome sequencing and reverse transcription-quantitative PCR (RT-qPCR) across decreasing low-micromolar to nanomolar O_2_ concentrations. As such, we could test the hypothesis that at micro- to nanomolar O_2_ concentrations, aerobic respiration is mediated by high-affinity TOs. Our data demonstrate that O_2_ concentrations down to the nanomolar level can be respired by low-affinity TOs, an unexpected physiological response, suggesting that the ability to respire O_2_ under micro- to nanooxic conditions is not exclusively based on the presence and activity of high-affinity TOs.

## RESULTS

### Distribution of low- and high-affinity terminal oxidases.

**FIG 1:**
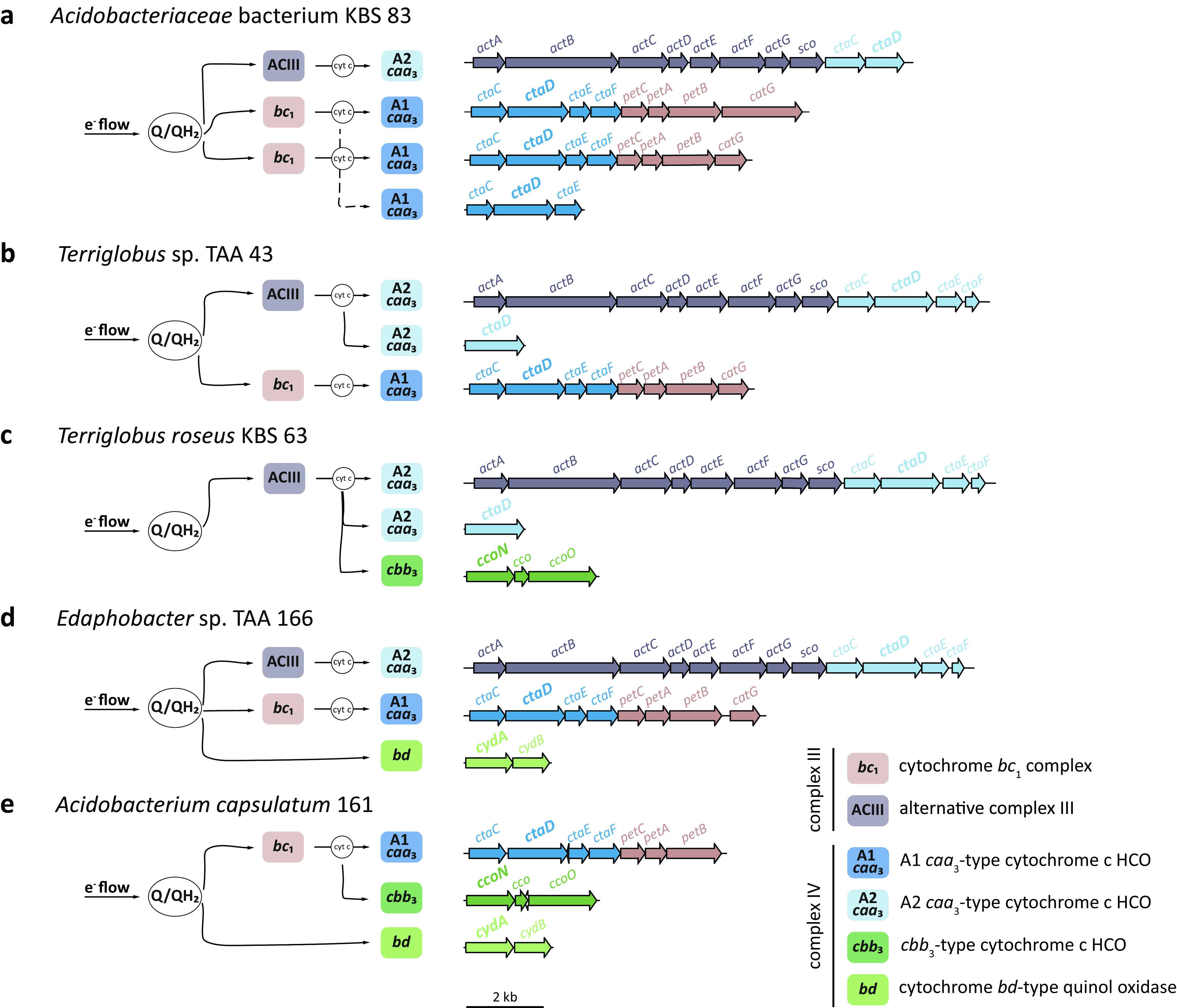
Schematic representation of electron (e^−^) flow in the predicted branched electron transport chains among the acidobacterial strains and organization of the respiratory genes in the respective genomes. The low- and high-affinity terminal oxidases of complex IV are depicted in blue and green, respectively. Complex III is depicted in gray (alternative complex III [ACIII]) or brown (cytochrome *bc*_1_ complex [*bc*_1_]). The quinone/quinol pools and cytochrome *c* are depicted as Q/QH_2_ and cyt c, respectively. The catalytic subunits of terminal oxidases are in boldface type. The dashed line in panel a indicates the electron flow via two possible *bc*_1_ complexes. Locus tags of the genes are listed in Data Set S1 in the supplemental material.

Five acidobacterial strains were chosen to explore their respiratory kinetics, and of these strains, three were chosen to explore their TO expression patterns across nanomolar O_2_ concentrations. All strains harbored branched respiratory chains terminating in multiple oxidases (Fig. 1; see also Data Set S1 in the supplemental material). They differed in their distributions of low- and high-affinity TOs (complex IV) as well as of complexes III (cytochrome *bc*_1_ complex and/or alternative complex III [ACIII]) (Fig. 1; Data Set S1).

10.1128/mSystems.00250-21.8 DATA SET S1 Genes encoding complex III and complex IV identified in the genomes of *Acidobacteriaceae* bacterium KBS 83, *Terriglobus* sp. TAA 43, Terriglobus roseus KBS 63, *Edaphobacter* sp. TAA 166, and Acidobacterium capsulatum 161. Download Data Set S1, XLSX file, 0.02 MB. Copyright © 2021 Trojan et al. 2021 Trojan et al. https://creativecommons.org/licenses/by/4.0/ This content is distributed under the terms of the Creative Commons Attribution 4.0 International license.

*Acidobacteriaceae* bacterium KBS 83 and *Terriglobus* sp. strain TAA 43 harbored multiple homologs of only low-affinity TOs; *Acidobacteriaceae* bacterium KBS 83 encoded three A1 *caa*_3_ HCOs and one A2 *caa*_3_ HCO (Fig. 1a), whereas *Terriglobus* sp. TAA 43 had one A1 *caa*_3_ HCO and two A2 *caa*_3_ HCOs encoded (Fig. 1b). Terriglobus roseus KBS 63 had two homologs of A2 *caa*_3_ HCOs (Fig. 1c), *Edaphobacter* sp. strain TAA 166 had one A1 *caa*_3_ HCO and one A2 *caa*_3_ HCO (Fig. 1d), and Acidobacterium capsulatum 161 had one A1 *caa*_3_ HCO encoded (Fig. 1e). In addition to low-affinity TOs, T. roseus KBS 63, *Edaphobacter* sp. TAA 166, and A. capsulatum 161 also harbored high-affinity TOs: *T. roseus* KBS 63 had a *cbb*_3_ type (C HCO) (Fig. 1c), *Edaphobacter* sp. TAA 166 had a *bd* type (Fig. 1d), and *A. capsulatum* 161 had both types (Fig. 1e).

There was consistent gene synteny for the A1 *caa*_3_ HCO, A2 *caa*_3_ HCO, C *cbb*_3_ HCO, and *bd*-type quinol oxidases and the adjacent complex III genes among the acidobacterial strains (Fig. 1). Genes for the A1 *caa*_3_ HCO were always located in an operon upstream of the genes encoding the *bc*_1_ complex (described here as a “superoperon”) (Fig. 1). The A2 *caa*_3_ HCO also occurred in a superoperon with the genes encoding ACIII, instead of the *bc*_1_ complex, and were located downstream of the ACIII genes (Fig. 1). Additional, single homologs of either the A1 or A2 *caa*_3_-type oxidases were detected in the genomes of *Acidobacteriaceae* bacterium KBS 83 (Fig. 1a), *Terriglobus* sp. TAA 43 (Fig. 1b), and *T. roseus* KBS 63 (Fig. 1c). *T. roseus* KBS 63 (Fig. 1c) and *A. capsulatum* 161 (Fig. 1e) contained *cbb*_3_ operons consisting of genes for *cbb*_3_ subunits N and O as well as an additional *cco* gene of unknown function. *Edaphobacter* sp. TAA 166 (Fig. 1d) and *A. capsulatum* 161 (Fig. 1e) contained both *cydA* and *cydB* subunits for the *bd*-type quinol oxidase.

### Assessment of O_2_ respiratory kinetics.

**FIG 2:**
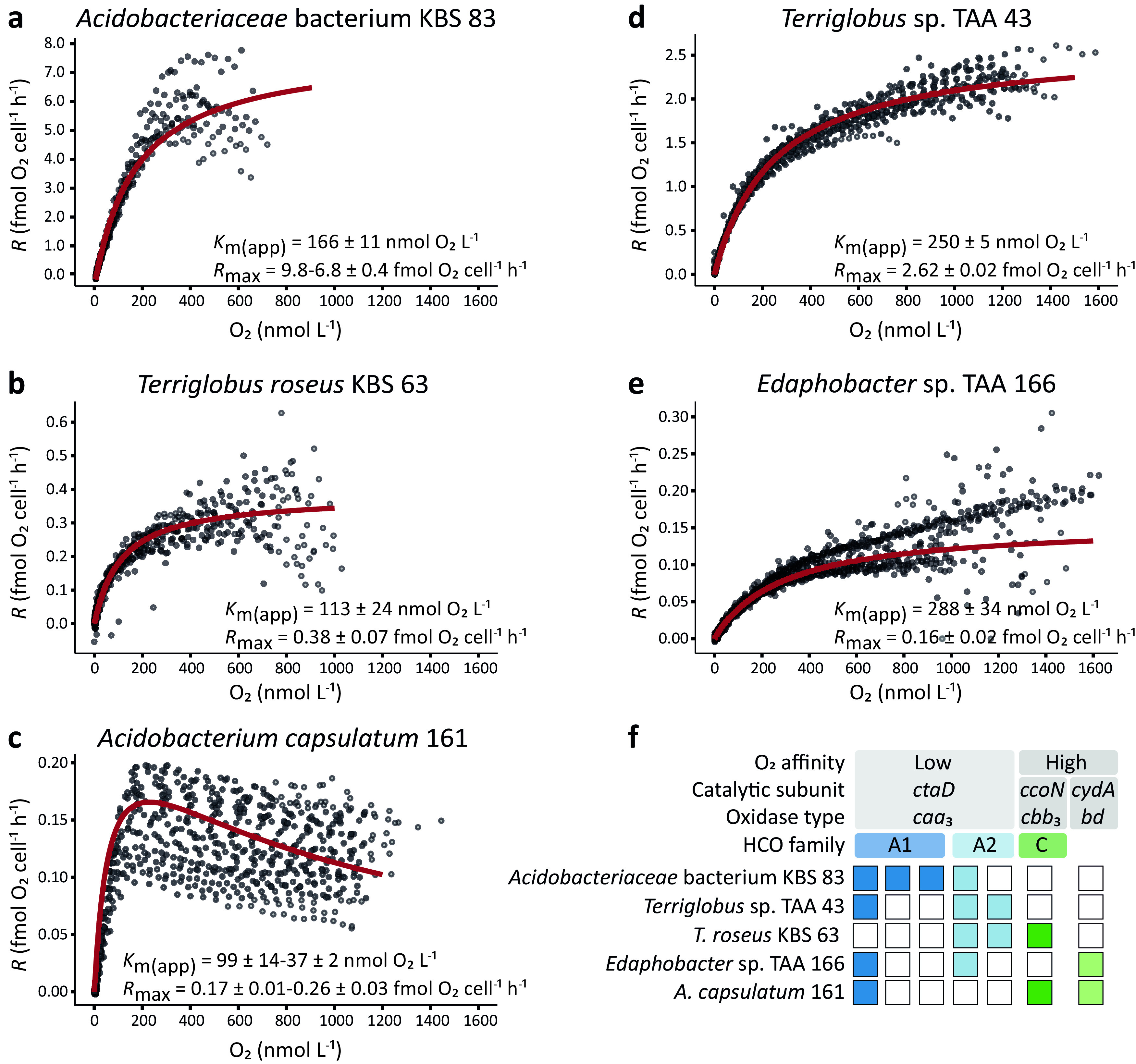
(a to e) Population respiratory kinetics of *Acidobacteriaceae* bacterium KBS 83 (a), *T. roseus* KBS 63 (b), *A. capsulatum* 161 (c), *Terriglobus* sp. TAA 43 (d), and *Edaphobacter* sp. TAA 166 (e). (f) Overview of the genomic identification of genes encoding terminal oxidases across the five acidobacterial strains. In panels a to e, the red curves indicate the Michaelis-Menten model best fit of the data. Across all strains, gray circles depict average respiration rates for biological triplicates over time, except for (i) *Acidobacteriaceae* bacterium KBS 83, where the gray circles represent the average respiration rates from biological duplicates, and (ii) *A. capsulatum* 161, where the individual replicates are depicted to illustrate the increase in respiration rates during the 24-h incubation period. See Tables S1 and S2 in the supplemental material for additional results of the temporal changes of kinetic parameters of *Acidobacteriaceae* bacterium KBS 83 and *A. capsulatum* 161, respectively. Apparent half-saturation constants (*K_m_*_(app)_) and maximum respiration rates (*R*_max_) are means ± standard errors.

We determined the O_2_ respiration rates and population apparent *K_m_* (*K_m_*_(app)_) values for the five acidobacterial strains with differing distributions of high- and low-affinity TOs in exponential phase (non-energy limited) with only O_2_-limiting respiration rates (Fig. 2). All strains followed Michaelis-Menten-type kinetics. *Acidobacteriaceae* bacterium KBS 83 and *Terriglobus* sp. TAA 43, both harboring only low-affinity TOs, had *K_m_*_(app)_ values for O_2_ of 166 ± 11 nmol O_2_ liter^−1^ (Fig. 2a) and 250 ± 5 nmol O_2_ liter^−1^ (Fig. 2d), respectively. The maximum population respiration rate (*V*_max_) of *Acidobacteriaceae* bacterium KBS 83 was on average 355 ± 12 nmol O_2_ liter^−1^ h^−1^, and the maximum respiration rates per cell (*R*_max_) progressively decreased over time from 9.8 to 6.8 ± 0.4 fmol O_2_ cell^−1^ h^−1^ (Fig. 2a; Table S1). The *V*_max_ of *Terriglobus* sp. TAA 43 was 998 ± 6 nmol O_2_ liter^−1^ h^−1^, and the *R*_max_ was constant at 2.6 ± 0.02 fmol O_2_ cell^−1^ h^−1^ (Fig. 2d).

10.1128/mSystems.00250-21.3 TABLE S1 Temporal development of kinetic parameters for the populations of *Acidobacteriaceae* bacterium KBS 83 cultures. *R*_max_ is corrected for cell aggregates of four cells as described in the text. Additional details on the calculations and modifications can be found in Materials and Methods and in Text S1 in the supplemental material. Download Table S1, DOCX file, 0.01 MB. Copyright © 2021 Trojan et al. 2021 Trojan et al. https://creativecommons.org/licenses/by/4.0/ This content is distributed under the terms of the Creative Commons Attribution 4.0 International license.

10.1128/mSystems.00250-21.1 TEXT S1 Supplementary materials and methods. Download Text S1, DOCX file, 0.04 MB. Copyright © 2021 Trojan et al. 2021 Trojan et al. https://creativecommons.org/licenses/by/4.0/ This content is distributed under the terms of the Creative Commons Attribution 4.0 International license.

10.1128/mSystems.00250-21.4 TABLE S2 Temporal development of kinetic parameters for the populations of Acidobacterium capsulatum 161 cultures. Download Table S2, DOCX file, 0.02 MB. Copyright © 2021 Trojan et al. 2021 Trojan et al. https://creativecommons.org/licenses/by/4.0/ This content is distributed under the terms of the Creative Commons Attribution 4.0 International license.

For *T. roseus* KBS 63 and *Edaphobacter* sp. TAA 166, harboring both low- and either a *cbb*_3_- or *bd-*type high-affinity TO, the *K_m_*_(app)_ values were 113 ± 24 nmol O_2_ liter^−1^ and 288 ± 34 nmol O_2_ liter^−1^, respectively (Fig. 2b and e). The *V*_max_ values of *T. roseus* KBS 63 and *Edaphobacter* sp. TAA 166 (201 ± 35 and 604 ± 69 nmol O_2_ liter^−1^ h^−1^, respectively) as well as their *R*_max_ values (0.38 ± 0.07 fmol O_2_ cell^−1^ h^−1^ and 0.16 ± 0.02 fmol O_2_ cell^−1^ h^−1^, respectively) were stable throughout the incubations (Fig. 2b and e). The *K_m_*_(app)_ value for *A. capsulatum* 161, harboring one low-affinity and both types of high-affinity TOs, decreased from 99 ± 14 to 37 ± 2 nmol O_2_ liter^−1^ (Table S2), with a final *K_m_*_(app)_ value 1 order of magnitude lower than the values of the other investigated strains (Fig. 2c). In addition, the *V*_max_ and *R*_max_ of *A. capsulatum* 161 progressively increased during the whole period of measurements from 2,150 ± 156 to 3,609 ± 430 nmol O_2_ liter^−1^ h^−1^ (Table S2) and from 0.17 ± 0.01 to 0.26 ± 0.03 fmol O_2_ cell^−1^ h^−1^, respectively (Fig. 2c). The respiration rates rose to a maximum as O_2_ concentrations increased and then descended to a nonzero asymptote. Additionally, the velocity curves saturated rapidly, compared to the other strains (Fig. 2e).

### Differential gene expression due to changing O_2_ concentrations.

Of the five strains, we selected three that encompass different combinations of low- and high-affinity TOs to compare changes in gene expression levels when exposed to different, decreasing O_2_ concentrations. Transcriptome analysis of *Acidobacteriaceae* bacterium KBS 83, *T. roseus* KBS 63, and *A. capsulatum* 161 showed that in the course of the time series, 5,121 (93% of all annotated genes), 4,239 (97%), and 3,321 (97%) genes, respectively, were transcribed at least at one time point across the O_2_ concentrations (Table S5).

10.1128/mSystems.00250-21.7 TABLE S5 RNA read numbers of the transcriptomes of *Acidobacteriaceae* bacterium KBS 83, *T. roseus* KBS 63, and *A. capsulatum* 161. Triplicate total RNA samples were sequenced on an Illumina NextSeq 550 high-output sequencer (75-nucleotide read length) after rRNA depletion using the NEB Ribo-Zero rRNA removal kit for bacteria. Download Table S5, DOCX file, 0.02 MB. Copyright © 2021 Trojan et al. 2021 Trojan et al. https://creativecommons.org/licenses/by/4.0/ This content is distributed under the terms of the Creative Commons Attribution 4.0 International license.

**FIG 3:**
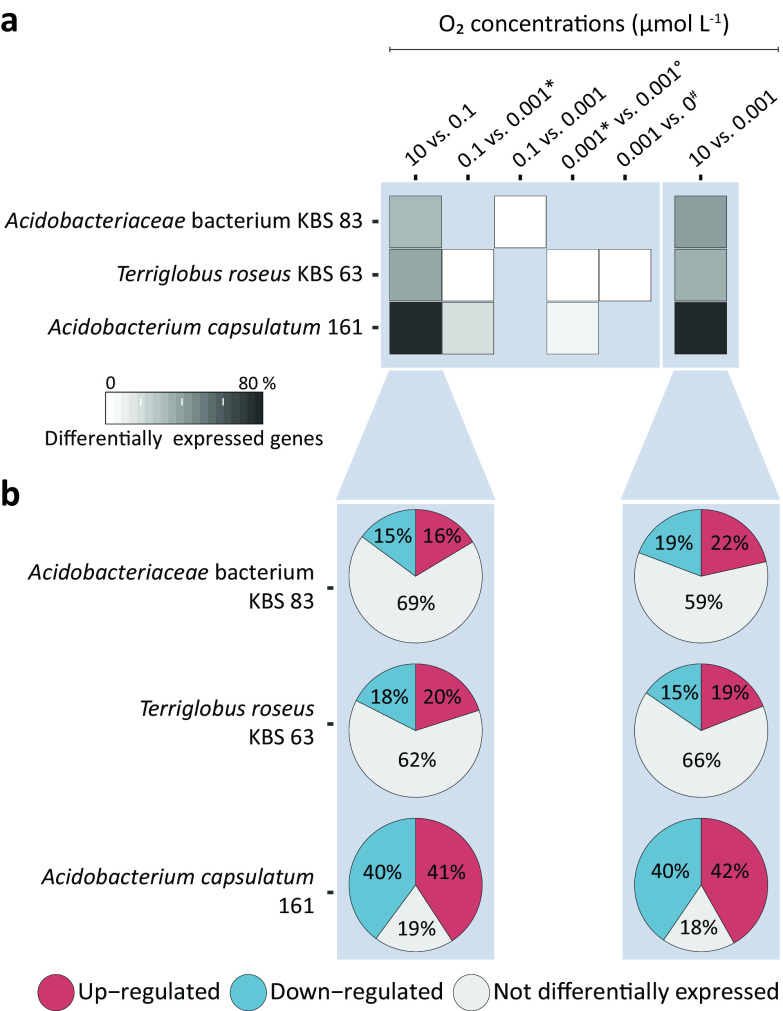
Impact of decreasing O_2_ concentrations on the transcriptomes of *Acidobacteriaceae* bacterium KBS 83, *T. roseus* KBS 63, and *A. capsulatum* 161. (a) Heat map depicting the proportions of genes that were differentially expressed (*P < *0.05) between two O_2_ concentrations (micromoles of O_2_ per liter). The darker the color, the higher the proportion of genes whose expression has significantly changed between two O_2_ concentrations. All comparisons were done after 60 min at each respective O_2_ concentration, with three exceptions: * depicts differential expression after 10 min, # depicts differential expression after 15 min, and ° depicts differential expression after 50 min. A concentration of 0.001 μmol O_2_ liter^−1^ is defined as apparent anoxia: O_2_ was still supplied (3.8 to 10.1 μmol O_2_ min^−1^) but could no longer be accurately determined. A concentration of 0 μmol O_2_ liter^−1^ indicates no O_2_ supply. (b) Breakdown of differentially expressed genes (*P < *0.05) for 10 versus 0.1 μmol O_2_ liter^−1^ and 10 versus 0.001 μmol O_2_ liter^−1^.

**FIG 4:**
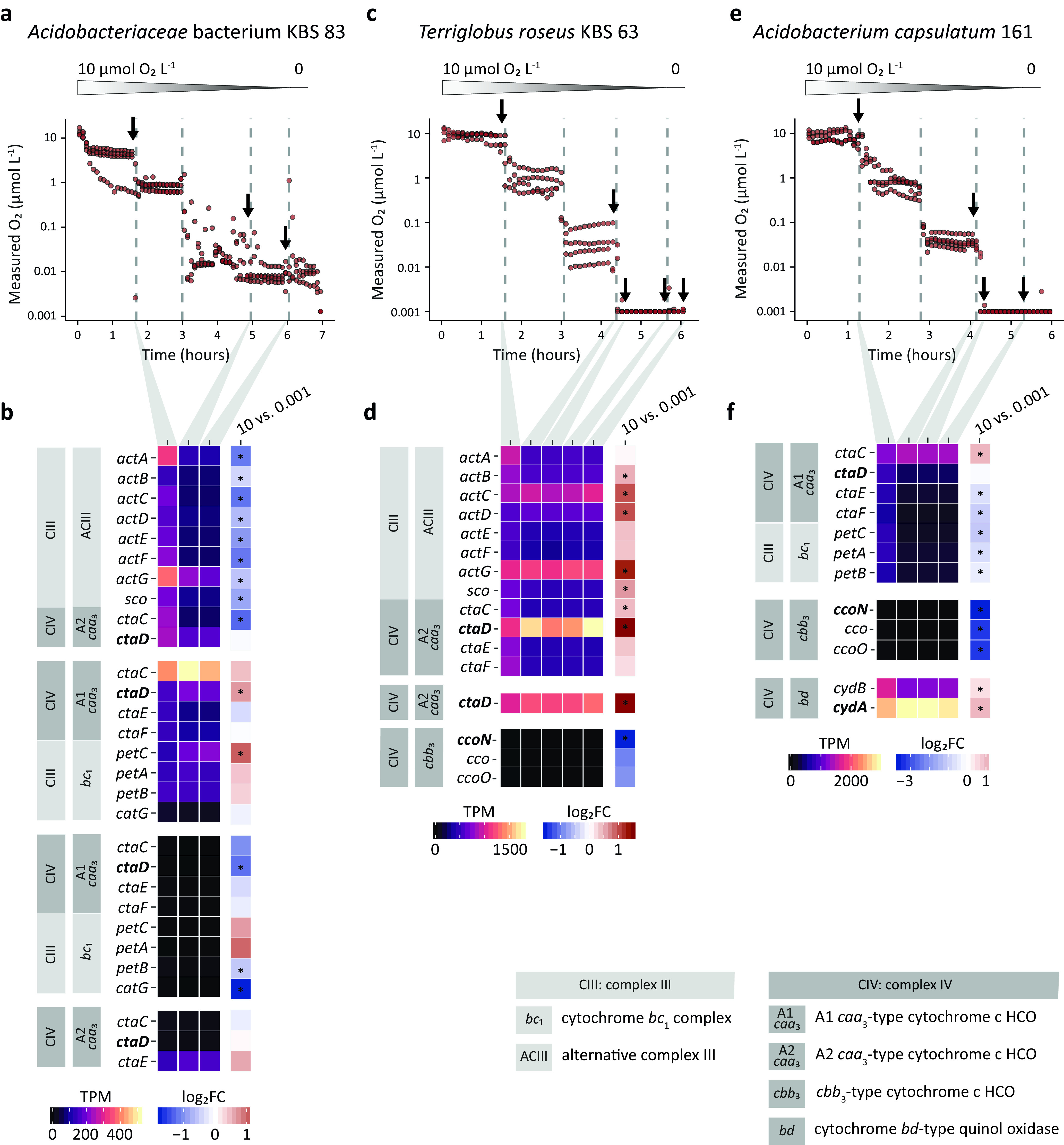
Respiration dynamics and transcription patterns of complex III and IV genes in the electron transport chain of *Acidobacteriaceae* bacterium KBS 83, *T. roseus* KBS 63, and *A. capsulatum* 161 exposed to decreasing O_2_ concentrations. (a, c, and e) Measured O_2_ concentrations in cultures of the three strains over time (*n* = 4 biological replicates/strain) during O_2_-limited incubations. O_2_ was decreased in a stepwise manner from 10 to 1 to 0.1 to 0.001 to 0 μmol O_2_ liter^−1^. Vertical dashed lines depict the transition time points, while arrows indicate transcriptome sampling points after 60, 10, or 15 min at the respective O_2_ concentrations. (b, d, and f) Time-resolved gene expression of complex III and complex IV at 10, 0.1, 0.001, and 0 μmol O_2_ liter^−1^. Heat maps show average transcript per million (TPM) values from biological replicates (*n* = 3). The last column depicts log_2_ fold changes (log_2_FC) of transcripts between 10 and 0.001 μmol O_2_ liter^−1^ after 60 min at the respective O_2_ concentrations. Downregulation is depicted in blue, and upregulation is in red. Asterisks depict significant differential expression (*P < *0.05). Catalytic subunits of terminal oxidases are in boldface type. A concentration of 0.001 μmol O_2_ liter^−1^ is defined as apparent anoxia: O_2_ was still supplied (3.8 to 10.1 μmol O_2_ min^−1^) but could no longer be accurately determined. A concentration of 0 μmol O_2_ liter^−1^ indicates no O_2_ supply. Data for all replicates, gene locus tags, and further details are listed in Data Set S2 in the supplemental material.

The decrease from 10 to 0.1 μmol O_2_ liter^−1^ had the greatest impact on the transcriptomes of all three strains, with the highest number of significantly differentially expressed genes observed (Fig. 3a). Among 1,602 (31%) differentially expressed genes of *Acidobacteriaceae* bacterium KBS 83, 16% were upregulated and 15% were downregulated upon the transition from 10 to 0.1 μmol O_2_ liter^−1^ after cells equilibrated for 60 min at each respective O_2_ concentration (Fig. 3b). For *T. roseus* KBS 63 and *A. capsulatum* 161, 38% (20% upregulated and 18% downregulated) and 81% (41% upregulated and 40% downregulated), respectively, were differentially expressed upon this transition from 10 to 0.1 μmol O_2_ liter^−1^ (Fig. 3b). Comparatively, there were few to no significant expression changes when transitioning from 0.1 to 0.001 μmol O_2_ liter^−1^ regardless of the equilibration time at the lower O_2_ concentration; similar patterns were observed in the transcriptome of *T. roseus* KBS 63 when transitioning from 0.001 to 0 μmol O_2_ liter^−1^ (Fig. 3a). The comparison between 10 and 0.001 μmol O_2_ liter^−1^ revealed the same overall transcription pattern as that for the transition from 10 to 0.1 μmol O_2_ liter^−1^ (Fig. 3). During these incubations, O_2_ was decreased in a stepwise manner from 10 μmol O_2_ liter^−1^ to anoxic conditions (<0.0005 μmol O_2_ liter^−1^) (Fig. 4). Below 0.01 μmol O_2_ liter^−1^, *Acidobacteriaceae* bacterium KBS 83, harboring only low-affinity TOs, consumed O_2_ at a respiration rate lower than the rate at which O_2_ was supplied, causing concentrations to never drop to anoxic conditions (Fig. 4a).

10.1128/mSystems.00250-21.9 DATA SET S2 Transcriptional changes of genes encoding the *bc*_1_ complex, alternative complex III, and complex IV of the electron transport chains of *Acidobacteriaceae* bacterium KBS 83, Terriglobus roseus KBS 63, and Acidobacterium capsulatum 161. Download Data Set S2, XLSX file, 0.03 MB. Copyright © 2021 Trojan et al. 2021 Trojan et al. https://creativecommons.org/licenses/by/4.0/ This content is distributed under the terms of the Creative Commons Attribution 4.0 International license.

In contrast, strains harboring both low- and high-affinity TOs (*T. roseus* KBS 63 and *A. capsulatum* 161) consumed all the supplied O_2_ at our lowest provided rate (i.e., 5.1 μmol O_2_ min^−1^ [*T. roseus* KBS 63] and 10.1 μmol O_2_ min^−1^ [*A. capsulatum* 161]). Their O_2_ uptake rates were higher than the O_2_ inflow rate, thereby creating an apparent anoxic environment below our detection limit of 0.0005 μmol O_2_ liter^−1^ (Fig. 4c and e).

### Transcriptional responses of branching electron transport chain key genes and terminal oxidases to decreasing O_2_ concentrations.

We further explored the transcriptional changes of TOs (complexes III and IV) of the ETC by focusing on key functional genes of these complexes (Fig. 4; Data Set S2).

### (i) *Acidobacteriaceae* bacterium KBS 83.

Continuous expression of two out of the four low-affinity *caa*_3_-type cytochrome *c* oxidases, one of the *bc*_1_-A1 *caa*_3_ superoperons and the ACIII-A2 *caa*_3_ superoperon, was observed across all investigated O_2_ concentrations, even after exposure to 0.001 μmol O_2_ liter^−1^ for an extended period of time (Fig. 4b; Data Set S2); similar patterns were observed by RT-qPCR (Fig. S1a). All genes of superoperon ACIII-A2 *caa*_3_ exhibited significantly lower expression levels at 0.001 than at 10 μmol O_2_ liter^−1^ (*P < *0.05), yet the catalytic subunit *ctaD* of the A2 HCO was consistently highly expressed across O_2_ concentrations and not significantly downregulated (Fig. 4b). In contrast, *ctaD* of the A1 HCO complex together with *petC* of the *bc*_1_ complex were significantly upregulated at 0.001 μmol O_2_ liter^−1^ (*P < *0.05). The transcription level of the electron-receiving subunit II (*ctaC*) was higher than that of the rest of the *bc*_1_-A1 *caa*_3_ superoperon and remained high upon transitions to lower O_2_ concentrations (Fig. 4b); the same responses were observed within the first 10 min after shifts of oxygenation by RT-qPCR (Fig. S1a). We still observed gene expression 15 min after the O_2_ supply was ceased (Fig. S1a). Even then, the O_2_ concentration did not fall below 0.01 μmol O_2_ liter^−1^ (Fig. 4a), and *Acidobacteriaceae* bacterium KBS 83 was still expressing its TOs after 3 h at 0.01 μmol O_2_ liter^−1^ (Fig. 4b). Of the other complexes IV, only *ctaE* that encodes subunit III of the single complex IV exhibited high expression levels (Fig. 4b).

10.1128/mSystems.00250-21.2 FIG S1 Relative gene expression of catalytic subunits of low- and high-affinity terminal oxidases in *Acidobacteriaceae* bacterium KBS 83 (a), *T. roseus* KBS 63 (b), and *A. capsulatum* 161 (c). Time-resolved relative expression levels of *ctaD*, *ccoN*, and *cydA* at 0.1, 0.001, and 0 μmol O_2_ liter^−1^ relative to the starting O_2_ concentration of 10 μmol O_2_ liter^−1^ are shown. Gene expression was normalized by transcripts of the *rpoB* reference gene. mRNA expression ratios (log_2_) were calculated compared to 10 μmol O_2_ liter^−1^. Samples for RT-qPCR were taken at several time points at the same O_2_ levels. Time is relative to stable O_2_ concentration readings as follows: 0, sampling as immediate as the Lumos reading indicated a stable signal after the switch to the new O_2_ concentration (equilibrium of O_2_ consumption by cells and N_2_-air gas inflow); 10, sampling after the reading was stable for 10 min; 60,  sampling after the reading was stable for 60 min; 15, sampling after 15 min of anoxia while cultures were purged with N_2_ gas. A concentration of 0.001 μmol O_2_ liter^−1^ is defined as apparent anoxia: O_2_ was still supplied (3.8 to 10.1 μmol O_2_ min^−1^) but could no longer be accurately determined. A concentration of 0 μmol O_2_ liter^−1^ indicates no O_2_ supply. The detection limit of Lumos was 0.0005 μmol O_2_ liter^−1^; hence, 0 μmol O_2_ liter^−1^ is <0.0005 μmol O_2_ liter^−1^. Error bars indicate standard errors from biological replicates (*n* = 4). Asterisks indicate significant changes (*P* < 0.05) compared to 10 μmol O_2_ liter^−1^ as determined by a pairwise fixed reallocation randomization test. HCO, heme-copper oxidase; nd, not detectable. See Table S4 in the supplemental material for further qPCR assay details. Download FIG S1, EPS file, 1.1 MB. Copyright © 2021 Trojan et al. 2021 Trojan et al. https://creativecommons.org/licenses/by/4.0/ This content is distributed under the terms of the Creative Commons Attribution 4.0 International license.

### (ii) *T. roseus* KBS 63.

The expression levels (transcripts per million [TPM]) of the catalytic subunit of the *cbb*_3_-type high-affinity TO (*ccoN*) across the investigated O_2_ concentrations were low (Fig. 4d; Data Set 2) and too low for reliable quantification by RT-qPCR (Fig. S1b). The catalytic subunits of both low-affinity A2 HCO TOs (*ctaD*) exhibited the highest expression levels and were transcribed at significantly higher levels (*P < *0.0001) at 0.001 than at 10 μmol O_2_ liter^−1^ (Fig. 4d). All other genes of the ACIII-A2 *caa*_3_ superoperon were also upregulated (Fig. 4b). After a shift to anoxic conditions, the single *ctaD* gene was still expressed and upregulated (Fig. S1b).

### (iii) *A. capsulatum* 161.

The *cbb*_3_-type high-affinity TO was transcribed at low levels at 10 μmol O_2_ liter^−1^ and was significantly downregulated (*P < *0.0001) at all subsequent lower concentrations (down to 0.001 μmol O_2_ liter^−1^) (Fig. 4f; Data Set S2). Expression of the *cbb*_3_-type high-affinity TO by RT-qPCR was seen from 10 to 0.1 μm O_2_ but only for 10 min at this concentration as measured by RT-qPCR (Fig. S1c). In contrast, the *bd*-type TO (*cydAB*) was expressed at all investigated O_2_ concentrations (10 to 0.001 μmol O_2_ liter^−1^). The relative abundance of *cydA* transcripts was very high under all O_2_ tensions (58-fold higher than that of the *rpoB* gene) (Data Set S2). RT-qPCR showed a clear and significant (*P *≤ 0.05) upregulation of the catalytic subunit *cydA* (Fig. S1c). Furthermore, *cydA* transcription levels were always high, even under anoxic conditions. The *ctaD* gene, encoding the catalytic subunit of the low-affinity A1 HCO, was continuously transcribed across all O_2_ concentrations as detected by transcriptomics and RT-qPCR (Fig. 4f; Fig. S1c). However, the proportion of *ctaD* transcripts decreased under anoxic conditions (Fig. 4f).

## DISCUSSION

Members of an abundant soil phylum, the *Acidobacteria*, respire environmentally relevant micro- and nanomolar O_2_ concentrations with the use of low-affinity TOs. Respiratory kinetics were determined using highly sensitive optical sensors, which allowed us to study the O_2_ kinetics with a high degree of accuracy. Our findings extend the current knowledge on O_2_ kinetics to species outside the *Proteobacteria*.

### Acidobacteria harbor branched respiratory chains terminating in multiple complexes IV with either low or high affinities for O_2_.

Branched ETCs terminating in differing terminal electron acceptors (such as O_2_, NO_3_, or NO_2_) are typically found in bacteria, providing flexibility when exposed to various environmental conditions (14, 15). Enzymatic redundancy in using a single electron acceptor (such as O_2_) can provide additional flexibility due to varying substrate affinities, allowing the microorganism to respire most efficiently across different concentrations, as seen in organisms living at the oxic-anoxic interface (28–34). This flexibility extends to our investigated soil acidobacterial strains, as many of them have branched ETCs that terminate in multiple complexes IV with either low or high affinities for O_2_ (Fig. 1). Furthermore, in select strains, genes for complex IV were detected in superoperons together with genes for complex III, either *bc*_1_ or alternative complexes III (Fig. 1), as previously seen in other members of the *Acidobacteria* and further phyla (35, 36), potentially functioning as respiratory supercomplexes (37–39). Although the physiological relevance of supercomplexes is still unclear (40), we suggest that this physical association might provide additional metabolic flexibility in the acidobacteria. The close association could allow a more favorable transfer between complexes, bypassing soluble electron carriers (39). Nevertheless, follow-up investigations will be needed to elucidate the advantage of the supercomplexes. The complex IV genes were also found independent from complex III genes in three strains (Fig. 1).

### The conventional high-affinity *cbb*_3_-type TO does not actively contribute to the capacity to respire O_2_ at nanomolar concentrations.

High-affinity TOs are historically believed to enable respiration and provide the capacity for energy conservation at trace concentrations of O_2_, a physiology that was shown to be widespread among bacteria and archaea of diverse environments, as suggested by genome surveys (16). Yet in the investigated acidobacterial strains, the *cbb*_3_-type high-affinity TO did not impart the capacity to respire O_2_ at nanomolar concentrations. In our experimental setup, strains harboring high-affinity TO genes had the potential to develop low apparent *K_m_* values by expressing these TO genes under O_2_-limited conditions, as in our incubations, the cells were exposed to multiple oxic-to-anoxic gradients over a 24-h period. Furthermore, our investigated strains harbor the minimal core, the CcoNO protein dyad (41), for the functionality of the enzyme (Fig. 1). Expression of the *cbb*_3_-type oxidase could not be detected below 10 μmol O_2_ liter^−1^ in both strains *T. roseus* KBS 63 and *A. capsulatum* 161 (Fig. 4), although they indeed consumed O_2_ down to (apparent) anoxia. Compared to reported *K_m_*_(app)_ values for O_2_ of *Proteobacteria* strains harboring *cbb*_3_-type oxidases measured by the same method (42), the *K_m_*_(app)_ value of *T. roseus* KBS 63 was high (113 nmol O_2_ liter^−1^) (Fig. 2b). This further provides evidence for the activity of the low-affinity oxidase(s) and suggests that it might be used for respiration in environments with low O_2_ concentrations, such as the heterogeneous soil environment. O_2_ fluctuations in soil are dynamic, and exposure to low-nanomolar O_2_ concentrations might be temporally limited to short intervals (43). Therefore, we hypothesize that the investment in the expression of a less-energy-efficient TO (the high-affinity *cbb*_3_ type) (44, 45) will not provide any competitive advantage for these investigated time intervals. At this time, it is unclear if the *cbb*_3_-type oxidase has lost its function to generate proton motive force in these strains. Alternatively, *cbb*_3_ TO expression in *T. roseus* KBS 63 and *A. capsulatum* 161 could be triggered by other factors, such as nutrient limitation or carbon depletion, as recently reported for Shewanella oneidensis (46).

### Utilization of acidobacterial *bd*-type oxidases at nanomolar O_2_ concentrations.

The *bd*-type oxidases are another type of high-affinity TO, which are less efficient at creating the charge gradient for ATP generation as they do not pump protons across the membrane but generate a proton motive force by transmembrane charge separation (12). Expression data showed a clear and significant upregulation of the catalytic subunit *cydA* gene as O_2_ concentrations decreased in *A. capsulatum* 161 (Fig. 4f; see also Fig. S1c in the supplemental material). This suggests that the *bd*-type oxidase contributed to the respiratory activity under trace O_2_ conditions. In contrast, the *cbb*_3_ type was transcribed only at low levels at 10 μmol O_2_ liter^−1^ and was significantly downregulated (*P* < 0.0001) at all subsequent lower concentrations (Fig. 4f; Fig. S1c). However, the use of the *bd*-type oxidase for respiration activity appears to be strain dependent. In another strain harboring a high-affinity *bd*-type oxidase (*Edaphobacter* sp. TAA 166), the expression of *cydA* could not be detected at any examined O_2_ concentration; rather, the low-affinity TOs were expressed across these O_2_ concentrations (RT-qPCR data not shown). Here, the *bd*-type oxidase could be contributing to physiological functions other than respiratory O_2_ reductions, such as reactive oxygen species (ROS) stress, iron deficiency, or nitric oxide stress responses (11, 12, 47).

Although the *bd*-type oxidases are not as efficient at creating a charge gradient, these oxidases have functional and structural characteristics that favor a faster electron flux than *cbb*_3_-type oxidases (11, 12), which could be advantageous under conditions with plentiful reducing potential stemming from carbon surplus. For instance, they receive electrons directly from the quinol pool and thereby take a shortcut through the branched ETC, bypassing any complexes III (Fig. 1). In support of this conjecture, *bd*-type oxidase genes were found to be more prevalent in environments where carbon is in excess, such as host-associated environments and carbon-rich forest soils compared to carbon-poor agricultural soils (16). As our investigated conditions were a combination of carbon surplus and O_2_ limitation, we therefore hypothesize that this selected for the utilization of the *bd*-type oxidase compared to the *cbb*_3_ type in *A. capsulatum* 161.

The strain expressing the *bd*-type oxidase under low O_2_ concentrations (*A. capsulatum* 161) was the only one that was inhibited by high O_2_ concentrations at its maximum respiration rate (*R*_max_) (Fig. 2c) (>250 nmol O_2_ liter^−1^). Furthermore, its *K_m_*_(app)_ value decreased over multiple oxic-anoxic shifts (*n* = 17) within 24 h, indicating a need for less substrate and, therefore, an adaptation to these conditions. This temporal kinetic development was previously observed for marine *Proteobacteria* (42). The final estimated *K_m_*_(app)_ value of *A. capsulatum* 161 (37 nmol O_2_ liter^−1^) suggests a mixed activity of low- and high-affinity TOs (Fig. 2c), with its high-affinity TO contributing a large portion of the *K_m_*_(app)_ value. This respiratory kinetic activity of *A. capsulatum* 161 suggests that this strain can use different O_2_ concentrations due to its enzymes’ O_2_ affinities. Presumably, this strain has a different strategy to exploit microoxic niches compared to the other investigated strains, which also could be advantageous in the soil when exposed to spatiotemporal gradients and diffusion limitations.

### Acidobacterial low-affinity TOs are used at nanomolar O_2_ concentrations.

Acidobacterial low-affinity *caa*_3_-type HCOs are functioning at previously unknown nanomolar O_2_ concentrations, as shown in the investigated strains (Fig. 4; Fig. S1). The use of low-affinity A HCOs at low concentrations of O_2_ is energetically favorable, as they have more free energy available for driving proton translocation due to poor O_2_ binding (44, 45) and a more efficient, and thus favorable, gating for proton leakage (44) than high-affinity TOs. High-affinity C HCOs typically exhibit higher catalytic activity at lower O_2_ concentrations due to a different redox-driven proton-pumping mechanism that allows an increased electron transfer rate and a faster reduction of O_2_ (48). Still, these high affinities come with a reduced proton-pumping efficiency (6, 44).

Many of the genes for the A2 *caa*_3_ HCO in *T. roseus* KBS 63 were not only expressed across varying O_2_ concentrations but in some cases also even upregulated at lower O_2_ concentrations (Fig. 4d; Fig. S1b). A continuous expression of low-affinity *caa*_3_-type TOs at low O_2_ concentrations was previously reported in aerobic marine bacterial species (19, 49); however, in that study (19), the high-affinity *cbb*_3_-type TO was upregulated at <0.2 μmol O_2_ liter^−1^. In our study, we did not observe any measurable contribution via transcriptomics or qPCR of the high-affinity *cbb*_3_-type TO in any of the strains at 10 to 0.001 μmol O_2_ liter^−1^, although we cannot completely rule out the possibility of a minor contribution (undetectable with our current methods) of the *cbb*_3_-type TO. Likewise, it is conceivable that high-affinity *cbb*_3_-type TOs function only at extremely low concentrations of O_2_ (<1 nmol O_2_ liter^−1^), which we currently cannot establish, maintain, and measure in the laboratory. Nevertheless, it appears that at the low O_2_ concentrations (down to 1 nmol O_2_ liter^−1^) investigated in this study, *T. roseus* KBS 63 definitely prioritizes the low-affinity TOs. The energetic advantage of the low-affinity TOs might explain the strategy of *T. roseus* KBS 63 to invest in the high expression and upregulation of A2 *caa*_3_ HCOs, compared to its *cbb*_3_-type high-affinity TO (Fig. 4d; Fig. S1b).

In contrast, *Acidobacteriaceae* bacterium KBS 83 harbored only low-affinity TOs (*caa*_3_ type) and was able to respire at O_2_ concentrations of 10 μmol O_2_ liter^−1^ and lower. Below 0.01 μmol O_2_ liter^−1^, it consumed O_2_ at a respiration rate lower than the rate at which O_2_ was supplied, causing concentrations not to reach anoxic conditions (Fig. 4a). However, complete consumption to anoxia was reached during the kinetics measurement experiments, reflecting the capacity to respire O_2_ at trace concentrations. This difference could be explained by a lower cell density in the incubations for transcriptome analysis, not allowing these incubations to reach anoxia during the time course of the incubations simply due to cell number. Alternatively, O_2_ diffusion could explain this discrepancy; this is unlikely as it was not observed in other incubations of the investigated acidobacteria. Efficient energy conservation (generating more ATP/electron) would be a vital survival strategy in times of substrate limitation in environments such as soil. It therefore might be an advantage to use low-affinity TOs even at nanomolar O_2_ concentrations as they, despite their lower reaction rate, ultimately drive more charges across the membrane per mole of O_2_, making them more efficient in energy conservation.

It appears that the capacity of *Acidobacteriaceae* bacterium KBS 83 to respire O_2_ under low concentrations was limited, as seen by the decreasing *V*_max_ and *R*_max_ over time (Table S1). Its *K_m_*_(app)_ value (166 nmol O_2_ liter^−1^) is lower than and in contrast to the previously reported *K_m_* value for the *caa*_3_-type oxidase of Pseudomonas aeruginosa (4,300 nmol O_2_ liter^−1^) (8) but in the same range as the one for the low-affinity cytochrome *bo*_3_ ubiquinol oxidase of Escherichia coli (200 nmol O_2_ liter^−1^) (5). Although it is difficult to compare *K_m_* values across studies as the determined *K_m_* values can differ dramatically depending on the applied approach (8, 50), we want to stress the fact that one has to be careful with historically set benchmarks that propagate in the literature. The determined *K_m_*_(app)_ values of our study represent ecophysiologically relevant estimates as we used whole populations and intact cells as well as highly sensitive optical sensors with an extremely low detection limit.

### Conclusion.

Microorganisms frequently have to cope with changing O_2_ tensions; therefore, having the flexibility to use a wide range of O_2_ concentrations is beneficial (16). Here, we show that members of a dominant and ubiquitous soil phylum (22, 24, 26), the *Acidobacteria*, have branched ETCs that terminate in multiple oxidases (high- and low-affinity TOs), providing them with respiratory flexibility and adaptability to environmental changes (14–16). More specifically, their low-affinity TOs are functioning at nanomolar O_2_ concentrations, presumably providing a great benefit for soil acidobacteria as they are more efficient in generating ATP than high-affinity TOs (44). We hypothesize that this strategy could be employed by other bacterial clades in soil as well as other habitats. Follow-up work is needed to ascertain if respiration at nanomolar O_2_ concentrations allows biomass production or population growth in the long run during exposure to such low O_2_ levels. In addition, low O_2_ concentrations and nutrient-rich conditions selected for the expression of the high-affinity *bd*-type oxidase rather than the *cbb*_3_ type, which presumably provides a more optimal balance of substrate oxidation and ATP production under these conditions. Follow-up studies are needed to elucidate the conditions under which acidobacterial *cbb*_3_-type TOs are employed for respiration. Our results extend the current knowledge on the respiratory flexibility of the prevalent *Acidobacteria*, which could help explain their success in the heterogeneous soil environment.

“Microaerobes” were previously defined as microorganisms that harbor high-affinity TOs in their genomes, either alone or in combination with low-affinity TOs, and use them to respire O_2_ in microoxic environments (16). However, “microoxic” or subatmospheric concentrations of O_2_ could be anything below 21% (vol/vol) O_2_, and within this range, the response of TOs can vary dramatically. In our study, we pushed microoxic to nanooxic conditions and explored the transcriptional response combined with enzyme kinetics to obtain a state-of-the-art assessment of their response to O_2_ tension. We detected high- and low-affinity TOs in multiple acidobacterial genomes and respiration at nanomolar O_2_ concentrations across the investigated strains. Yet our gene expression data did not indicate any detectable contribution of the *cbb*_3_-type high-affinity TOs at these O_2_ concentrations; only one strain had contributions from the high-affinity *bd*-type TO. This suggests that the capability for microaerobic respiration in these acidobacteria is not solely due to the presence and associated activity of high-affinity TOs. Instead, the acidobacterial microaerobic lifestyle seems to also be imparted by low-affinity *caa*_3_-type TOs that enable them to respire O_2_ at nanomolar concentrations. This illustrates that the presence of a high-affinity TO in a genome is not a prerequisite for microaerobic respiration. To that end, we would like to amend the definition of microaerobe to encompass microorganisms that are capable of respiring O_2_ under microoxic conditions via the utilization of high- or low-affinity TOs. Furthermore, these findings demonstrate that it can be challenging to make predictions on the ecophysiology and lifestyle of microorganisms based solely on their genomic information, even for a process as well studied as aerobic respiration.

## MATERIALS AND METHODS

### Strains and growth conditions.

Five chemoorganotrophic strains of the family *Acidobacteriaceae*, *Acidobacteriaceae* bacterium KBS 83 (DSM 24295), *Terriglobus* sp. TAA 43 (LMG 30954; DSM 24187), Terriglobus roseus KBS 63 (NRRL B-41598^T^; DSM 18391), *Edaphobacter* sp. TAA 166 (LMG 30955; DSM 24188), and Acidobacterium capsulatum 161 (ATCC 51196; DSM 1124), were grown in vitamins and salts base (VSB) medium (51, 52) amended with 10 mM glucose as the sole carbon source at pH 6 or 5 (*A. capsulatum* 161). Additional information on the strains was reported previously (27, 52–54).

### Setup and incubation for respiratory kinetic parameters.

The details of the setup and experimental procedure were previously described (34, 42, 55). Briefly, the incubations were conducted in custom-made 500- or 1,100-ml glass bottles, which had been sequentially rinsed with a solution containing 0.1 M NaOH, 0.1 M HCl, and autoclaved water to prevent contamination. A continuous flow of N_2_ was maintained while filling the bottles with N_2_-purged medium and subsequent sealing with ground-glass stoppers. Exponential-phase acidobacterial cells were injected into these bottles (2 to 3 replicates/strain), while glass-coated magnetic stirrers homogenized the suspension. The O_2_ concentration was optically determined every 20 s by luminescence-based O_2_ sensors (Lumos) with sensor spots (measurement range, 0.5 to 1,500 nmol O_2_ liter^−1^) (56) glued onto the inside of the bottles. Bottles were incubated at room temperature and shielded from light for 24 h. Air-saturated water (4 to 5 ml) was repeatedly injected into the bottles after anoxia was reached by cell respiration, with peak concentrations ranging from 600 to 1,620 nmol O_2_ liter^−1^. One milliliter of the cell suspension was collected and fixed with 1% glutaraldehyde (Sigma-Aldrich, St. Louis, MO, USA) to determine cell numbers as described previously (42). After the incubations were completed, O_2_ sensors were calibrated with oxygenated water and sodium dithionite.

### Calculation of kinetic parameters.

O_2_ consumption rates were calculated from linear regression of O_2_ concentrations over time in intervals of 6 min from the highest O_2_ concentration down to anoxia. Kinetic parameters, the apparent half-saturation constant (*K_m_*_(app)_) and the maximum respiration rate (*V*_max_) of the Michaelis-Menten equation, were estimated by performing nonlinear parametric fits on the respiration-versus-O_2_-concentration curves for each replicate. *V*_max_ and *K_m_*_(app)_ were varied iteratively until the best fit was obtained by least-square fits using Solver in Microsoft Excel (57). Maximum respiration rates per cell (*R*_max_) were calculated by dividing the population respiration rate (*V*_max_) by cell numbers. Michaelis-Menten plots of respiration rates versus O_2_ concentrations were obtained by fitting a Michaelis-Menten model to the data using the equation *V* = (*V*_max_ × [O_2_]) × (*K_m_* + [O_2_])^−1^, where *V* is the rate, *V*_max_ is the maximum rate (nanomoles of O_2_ per liter per hour), *K_m_* is the half-saturation constant (nanomoles of O_2_ per liter), and [O_2_] is the substrate concentration (nanomoles of O_2_ per liter). Additional modifications of the Michaelis-Menten equation and further corrections can be found in Text S1 (Supplemental Materials and Methods 1) and Tables S1 and S2 in the supplemental material.

### Transcriptional profiling incubations.

*Acidobacteriaceae* bacterium KBS 83, *T. roseus* KBS 63, and *A. capsulatum* 161 were grown in biological quadruplicates in glass bottles (Schott) containing 1 liter of VSB minimal medium amended with 10 mM glucose under fully aerated conditions. Once cells reached exponential phase, they were transferred into HCl-sterilized and autoclaved-water-rinsed glass bottles equipped with internally preglued sensing spots. Incubations were run for 225 min and split into four discrete, declining O_2_ concentrations (10 μmol O_2_ liter^−1^, 1 μmol O_2_ liter^−1^, 0.1 μmol O_2_ liter^−1^, and 0.001 μmol O_2_ liter^−1^) down to anoxia (0 μmol O_2_ liter^−1^ is <0.0005 μmol O_2_ liter^−1^) obtained by purging with N_2_-air mixtures (Table S3). O_2_ concentrations were monitored by two Lumos systems with different sensitivity ranges (0.5 to 1,500 and 10 to 20,000 nmol O_2_ liter^−1^) (56). At every time point (Table S3), 30 to 50 ml of the culture was collected for RNA extractions by syringes prefilled with a phenol-stop solution (58). The sensor spots were calibrated after the incubations with oxygenated water and sodium dithionite. Additional details can be found in Text S1 (Supplemental Materials and Methods 2).

10.1128/mSystems.00250-21.5 TABLE S3 Sampling scheme of transcriptional profiling incubations of acidobacterial cultures (*Acidobacteriaceae* bacterium KBS 83, *Terriglobus* sp. TAA 43, Terriglobus roseus KBS 63, *Edaphobacter* sp. TAA 166, and Acidobacterium capsulatum 161) under declining oxygen concentrations. Download Table S3, DOCX file, 0.01 MB. Copyright © 2021 Trojan et al. 2021 Trojan et al. https://creativecommons.org/licenses/by/4.0/ This content is distributed under the terms of the Creative Commons Attribution 4.0 International license.

### RNA extraction and purification.

Total RNA was extracted from frozen cell pellets using an acidic phenol-chloroform–isoamyl alcohol protocol as described previously (59), with mechanical disruption (FastPrep-24 bead beater; MP Biomedicals, Heidelberg, Germany). The extraction supernatant was purified using standard chloroform-isoamyl alcohol purification, and RNA was precipitated using a polyethylene glycol (PEG) solution and RNA-grade glycogen by centrifugation (21,130 × *g* for 1 h at 4°C). Coextracted DNA was digested using a Turbo DNA-free kit (Thermo Fisher), and complete DNA removal was verified by failure to obtain quantitative PCR (qPCR) amplification products with the purified RNA template, targeting the *rpoB* gene encoding the β subunit of the DNA-directed RNA polymerase, under the qPCR conditions described in Table S4. A more detailed protocol can be found in Text S1 (Supplemental Materials and Methods 3).

10.1128/mSystems.00250-21.6 TABLE S4 Target genes and RT-qPCR details for primers developed in this study. Download Table S4, DOCX file, 0.02 MB. Copyright © 2021 Trojan et al. 2021 Trojan et al. https://creativecommons.org/licenses/by/4.0/ This content is distributed under the terms of the Creative Commons Attribution 4.0 International license.

### Primer design, cDNA synthesis, RT-qPCR, and data analysis.

Specifications of the newly designed primers targeting the catalytic subunits (subunit I) of the TOs are listed in Table S4. See Text S1 (Supplemental Materials and Methods 4) for details on primer design, cDNA synthesis, reverse transcription-qPCR (RT-qPCR), and data analysis.

### Transcriptome sequencing.

Triplicate total RNA samples of *Acidobacteriaceae* bacterium KBS 83, *T. roseus* KBS 63, and *A. capsulatum* 161 from selected O_2_ concentrations and time points were sent to the Vienna BioCenter Core Facilities. rRNA was depleted using the New England BioLabs (NEB) Ribo-Zero rRNA removal kit for bacteria. Sequencing was performed on an Illumina NextSeq 550 system, resulting in a total of 36 samples with 8.2 million to 18.2 million 75-nucleotide reads each.

### Transcriptome data processing and statistical analyses.

Raw reads were trimmed of sequencing adapters and low-quality 3′ ends using BBduk (BBtools v37.61; https://jgi.doe.gov/data-and-tools/bbtools/) with default parameters and error corrected using the Bayes-Hammer module of SPAdes assembler version 3.13.0 (60). Any reads mapping to either SILVA small-subunit (SSU) or large-subunit (LSU) release 132 (61) or the 5S rRNA database (62) with a sequence identity of >70% (performed with BBmap and BBtools; https://jgi.doe.gov/data-and-tools/bbtools/) were removed from the data set. The remaining reads were mapped to the publicly available genomes of the acidobacterial strains (53). The RNA reads per gene were summarized using the featureCounts tool from the Subread package v1.6.2 (63). Based on the generated read count tables, transcripts per million were calculated in R v3.6.0. Differential expression analyses, such as calculations of log_2_ fold changes of relative transcript abundances and the significance of these changes, were performed in DESeq2 v1.26.0 using default parameters and a *P* value cutoff of 0.05 (64).

### Data availability.

The raw transcriptomic reads are available under BioProject accession number PRJNA635786. The code and pipelines used for data analysis are available upon request.
