# Supplementary material for: Microaerobic Lifestyle at Nanomolar O2 Concentrations Mediated by Low-Affinity Terminal Oxidases in Abundant Soil Bacteria
Source: mSystems. 2021 Jul 6;6(4):e00250-21. doi: 10.1128/mSystems.00250-21 (PMC8407424; doi:10.1128/mSystems.00250-21)
Supplement: TABLE S1 [file msystems.00250-21-st001.docx]

|  | | | | | | |
| --- | --- | --- | --- | --- | --- | --- |
| **Time  (hours)** | ***V_max_***  **(nmol O_2_ L^-1^ h^-1^)** | **SE** | ***K_m_* (nmol L^-1^)** | **SE** | ***R_max_***  **(fmol O_2_ cell^-1^ h^-1^)** | **SE** |
| 4.3 | 370.25 |  | 165.7 |  | 9.8 |  |
| 8.9 | 350.04 | 11.3 | 165.7 | 10.7 | 8.3 | 0.3 |
| 14.3 | 342.92 | 18.3 | 165.7 | 10.7 | 7.2 | 0.4 |
| 19.6 | 357.09 | 16.7 | 165.7 | 10.7 | 6.8 | 0.3 |
| *V*_max_ = maximum respiration rate; *K*_m_ = apparent half-saturation Michaelis-Menten constant; *R*_max_ = maximum respiration rate; biological replicates = 2; oxic/anoxic shifts = 4; SE = standard error. | | | | | | |
