# Supplementary material for: Microaerobic Lifestyle at Nanomolar O2 Concentrations Mediated by Low-Affinity Terminal Oxidases in Abundant Soil Bacteria
Source: mSystems. 2021 Jul 6;6(4):e00250-21. doi: 10.1128/mSystems.00250-21 (PMC8407424; doi:10.1128/mSystems.00250-21)
Supplement: TEXT S1 [file msystems.00250-21-s0001.docx]

**Supplemental Material**

**Microaerobic lifestyle at nanomolar O_2_ concentrations mediated by low-affinity terminal oxidases in abundant soil bacteria**

Daniela Trojan, Emilio García-Robledo, Dimitri V. Meier, Bela Hausmann, Niels Peter Revsbech, Stephanie A. Eichorst, Dagmar Woebken

Address correspondence to Stephanie A. Eichorst, [stephanie.eichorst@univie.ac.at](mailto:stephanie.eichorst@univie.ac.at)

**Supplementary Materials and Methods**

**Supplemental Materials and Methods-1. Calculation of kinetic parameters.**

*Acidobacteriaceae* bacterium KBS 83 is typically observed in aggregates of at least four cells, tending to form even bigger flocs visible to the naked eye, which was also previously reported (1). As such, we corrected our estimates accordingly and divided its respiration rates per cell (*R*_max_) by four, and the best fit curve in **Figure 2** represents the mean best fit to the initial respiration rate (see **Table S1** for all data). We believe the morphological characteristics did not substantially affect our kinetic measurement results, as the affinity for O_2_ of *Terriglobus* sp. TAA 43, a strain that is neither known to form cell aggregates and nor is it harboring any high-affinity terminal oxidases, had a comparably high *K*_m(app)_. The data of *T. roseus* KBS 63 did not fit to a standard Michaelis-Menten equation as maximum respiration rates increased linearly with higher O_2_ concentrations. The presence of reduced compounds reacting with O_2_ could explain the deviation and thus an additional linear O_2_-consuming reaction was taken into account: *V* = ((*V*_max_ × [O_2_]) × (*K*_m_ +[O_2_])^-1^ + *K_1_* × [O_2_]) where *K_1_* is the linear rate constant (*K*_1_ = 0.3 ± 0.06 h^-1^). The data of *A. capsulatum* 161 were modeled by accounting for O_2_ inhibition at high concentrations using the following deviation from the Michaelis-Menten equation: *V* = (*V*_max_ × [O_2_]) × (*K*_m_ + [O_2_] × (1+[O_2_]/*K_s_*))^-1^, where *K_s_* is the dissociation constant. The best fit curve in **Figure 2** represents the mean best fit over time (see **Table S2** for all data).

**Supplemental Materials and Methods-2. Setup and time course transcriptional profiling incubations.**

For microoxic incubations, four strains, *Acidobacteriaceae* bacterium KBS 83, *T. roseus* KBS 63, *Edaphobacter* sp. TAA 166 and *A. capsulatum* 161 were grown up in biological quadruplicates in glass bottles (Schott) in 1 L of minimal media VSB-6 and VSB-5, respectively, amended with 10 mM glucose. Growth in surplus nutrition allowed us to assume that O_2_ was the only key effector regulating gene expression. Additionally, we ensured complete aeration and adaptation to the experimental conditions by vigorously bubbling with 0.2 µm-filtered compressed air during growth of the bacterial cultures. This consideration allowed us to assume that we established microoxic conditions only at the start of our measurements. Contamination controls (10 mM glucose-amended VSB-6 media, n = 3) were started in parallel and no microbial growth was observed in any bottle. Once cells reached exponential phase, indicated by cell density and culture turbidity, the microoxic incubations were started by transferring the culture into HCl-sterilized and autoclaved-water-rinsed glass bottles equipped with internally pre-glued sensing spots. The butyl rubber caps allowed for bubbling with a 0.2 µM-filtered N_2_-air gas mixture (99.998 vol %; Air Liquide, Paris, France) by a long glass tube that penetrated the cap and reached to the very bottom of the bottle. A glass air stone together with a glass-coated magnetic stirrer ensured homogeneous aeration and mixing of incubated cultures. The gas flow and mixing ratio of N_2_ and compressed air was adjusted by a mass flow controller (Brooks instruments, Hatfield, USA). The gas outlet was arranged through a hypodermic needle and a second sampling glass tube allowed biomass collection throughout the incubation. The highly sensitive LUMOS system (measuring range 0.5 to 1500 nmol O_2_ L^-1^) was complemented by a second LUMOS system with platinum sensing spots of a measuring range of 10 to 20 000 nmol O_2_ L^-1^ (2) to monitor O_2_ concentration throughout the incubations. The gas flow (200 mL min ^-1^) was split among the replicates and allowed for simultaneous gas-bubbling of two bottles; the four independent biological replicates were thus successively incubated in pairs of two. Incubations were run for 225 minutes and split into four discrete, declining O_2_ concentrations (10 µmol O_2_ L^-1^, 1 µmol O_2_ L^-1^, 0.1 µmol O_2_ L^-1^, 0.001 µmol O_2_ L^-1^) down to anoxia (0 µmol O_2_ L^-1^ is < 0.0005 µmol O_2_ L^-1^) obtained by N_2_-purging (instead of using the N_2_-air mixture) (**Table S3**). 0.001 µmol O_2_ L^-1^ represents an “apparent anoxia” as this concentration is close to the detection limit and could not be measured anymore although O_2_ was still provided: 3.8 µmol O_2_ min^-1^ for *Acidobacteriaceae* bacterium KBS 83, 5.1 µmol O_2_ min^-1^ for *T. roseus* KBS 63, 5.1 µmol O_2_ min^-1^ for *Edaphobacter* sp. TAA 166 and 10.1 µmol O_2_ min^-1^ for *A. capsulatum* 161. At every timepoint (**Table S3**), 30 ml (*T. roseus* KBS 63, *Edaphobacter* sp. TAA 166 and *A. capsulatum* 161) or 50 ml (*Acidobacteriaceae* bacterium KBS 83) of culture were collected for RNA extraction by a syringe connected to the sampling glass tube. For an immediate inactivation, the syringes were pre-filled with an acidic phenol-stop solution (3) (95% EtOH, 5% TRIzol Reagent (ThermoFisher Scientific, Waltham, USA) and pre-cooled at 4°C. Samples were transferred to conical tubes and centrifuged at 4 °C (12 000 x *g*, 10 minutes). The supernatant was discarded, cell pellets were snap frozen in liquid nitrogen and then stored at –80 °C. The sensor spots were calibrated after the incubations with oxygenated water and sodium dithionite.

**Supplemental Materials and Methods-3. RNA extraction and purification.**

Frozen cell pellets were re-suspended in a phosphate buffer (pH 5.8) and disrupted in a Lysing Matrix E tube (MP Biomedicals, Heidelberg, Germany) in the presence of TNS (Tris-HC, NaCl, sodium dodecyl sulfate) extraction buffer (4) and phenol/chloroform/isoamyl alcohol (125:24:1; pH 4.5; Sigma-Aldrich, St. Louis, USA) using a FastPrep-24 bead beater (30 s, 4 ms^-1^) and the CoolPrep Cryogenic adapter (MP Biomedicals). The extraction supernatant was then purified using a standard chloroform/isoamyl alcohol purification, and RNA was precipitated using a PEG solution (30% polyethylene glycol, 1.6 M NaCl) and RNA-grade glycogen (20 mg mL^-1^; ThermoFisher Scientific, Waltham, USA) for 1 hour at 4 °C and subsequent centrifuging (21130 x *g*, 1 hour, 4 °C), washed twice at 4 °C with cooled 70% EtOH, re-suspended in nuclease-free water, aliquoted, and stored at -80 °C. Co-extracted DNA was quantified using Quant-iT PicoGreen dsDNA assay kit (ThermoFisher Scientific) as per manufacturer's instructions. DNA was digested using TURBO DNA-*free* kit (ThermoFischer) and the RNA was purified by EtOH precipitation using RNAse-free sodium acetate (3 M, pH 5.5). Complete DNA removal was verified by failure to obtain qPCR amplification products with the purified RNA template, targeting the *rpo*B gene using the RT-qPCR conditions described below. RNA yields were quantified using Quant-iT RiboGreen RNA assay kit (ThermoFischer).

**Supplemental Materials and Methods-4. Primer design, cDNA synthesis, reverse-transcriptase quantitative PCR (RT-qPCR) and data analysis.**

Geneious v9.1.4 (5) was used for primer design. For all assays, annealing- and fluorescence detection temperature were 68 °C and 80 °C, respectively. Specifications of the newly designed, strain- and gene-specific primers targeting the catalytic subunits (subunit I) of the TOs and the DNA-directed RNA polymerase β subunit (reference gene) are listed in **Table S4** together with the newly developed RT-qPCR assays for relative quantification. Complementary DNA (cDNA) was synthesized from RNA using SuperScript IV Reverse Transcriptase (ThermoFischer) using 50 ng purified RNA and 50 ng μl^−1^ of provided random hexamer primers, as described by the manufacturer with a prolonged incubation time of annealed RNA and RT reaction mix of 120 minutes. cDNA products were diluted prior to RT-qPCR (1:4.5 (*Acidobacteriaceae* bacterium KBS 83); 1:12 (*T. roseus* KBS 63); 1:15 (*Edaphobacter* sp. TAA 166); 1:10 (*A. capsulatum* 161). 5 µL of diluted cDNA were inserted in the 20 µL iQ SYBR Green Supermix (Bio-Rad Laboratories GmbH, Feldkirchen, Germany) reaction including a final concentration of 0.1 µg µL^-1^ bovine serum albumin. Biological quadruplicates were run in technical triplicates and inter-run calibrators were included on each plate in order to detect and correct for run-to-run variations.

**qPCR data analysis:** the Relative Expression Software Tool–Multiple Condition Solver (REST-MCS © version 2) (6, 7) was used for data analysis and statistical testing. Expression of the target genes across the different O_2_ conditions was compared to the reference condition (10 µmol O_2_ L^-1^). Significance of the ratio results were tested using a pair-wise fixed reallocation randomization test implemented in REST-MCS ©. Expression of *rpoB* was used for normalization and data were plotted as log_2_ ratios.

**Supplementary References**

1. S. A. Eichorst, C. R. Kuske, T. M. Schmidt, Influence of plant polymers on the distribution and cultivation of bacteria in the phylum *Acidobacteria*. *Appl. Environ. Microbiol.* **77**, 586–596 (2011).

2. P. Lehner, *et al.*, LUMOS - a sensitive and reliable optode system for measuring dissolved oxygen in the nanomolar range. *PLoS One* **10**, e0128125 (2015).

3. K. D. Kits, M. G. Klotz, L. Y. Stein, Methane oxidation coupled to nitrate reduction under hypoxia by the Gammaproteobacterium *Methylomonas denitrificans*, sp. nov. type strain FJG1. *Environ. Microbiol.* **17**, 3219–3232 (2015).

4. R. Angel, Total nucleic acid extraction from soil. *Protoc. Exch.*, e-pub ahead of print 23 October 2012;doi:10.1038/p (2012).

5. M. Kearse, *et al.*, Geneious Basic: an integrated and extendable desktop software platform for the organization and analysis of sequence data. *Bioinformatics* **28**, 1647–1649 (2012).

6. M. W. Pfaffl, G. W. Horgan, L. Dempfle, Relative expression software tool (REST) for group-wise comparison and statistical analysis of relative expression results in real-time PCR. *Nucleic Acids Res.* **30**, e36 (2002).

7. M. W. Pfaffl, A new mathematical model for relative quantification in real-time RT-PCR. *Nucleic Acids Res.* **29**, e45 (2001).
