# Supplementary material for: Microaerobic Lifestyle at Nanomolar O2 Concentrations Mediated by Low-Affinity Terminal Oxidases in Abundant Soil Bacteria
Source: mSystems. 2021 Jul 6;6(4):e00250-21. doi: 10.1128/mSystems.00250-21 (PMC8407424; doi:10.1128/mSystems.00250-21)
Supplement: TABLE S2 [file msystems.00250-21-st002.docx]

|  | | | | | | | |
| --- | --- | --- | --- | --- | --- | --- | --- |
| **Time  (hours)** | ***V*_max_**  **(nmol O_2_ L^-1^ h^-1^)** | **SE** | ***K*_m_**  **(nmol O_2_ L^-1^)** | **SE** | ***R*_max_**  **(fmol O_2_ cell^-1^ h^-1^)** | **SE** | ***K*_s_*/K*_m_** |
| 0.1 | 2150 | 156 | 99 | 14 | 0.17 | 0.01 | 8 |
| 1.5 | 2496 | 128 | 93 | 5 | 0.19 | 0.01 | 9 |
| 2.9 | 2698 | 80 | 101 | 19 | 0.21 | 0.01 | 8 |
| 5.1 | 2943 | 66 | 88 | 8 | 0.23 | 0.01 | 9 |
| 6.5 | 3368 | 109 | 94 | 10 | 0.26 | 0.01 | 9 |
| 7.9 | 3318 | 267 | 78 | 13 | 0.26 | 0.02 | 10 |
| 9.3 | 3695 | 86 | 79 | 11 | 0.28 | 0.01 | 10 |
| 10.8 | 3823 | 346 | 92 | 8 | 0.29 | 0.03 | 9 |
| 12.3 | 4161 | 191 | 95 | 9 | 0.32 | 0.01 | 9 |
| 13.8 | 3862 | 368 | 55 | 4 | 0.30 | 0.03 | 15 |
| 15.3 | 3715 | 99 | 40 | 1 | 0.29 | 0.01 | 20 |
| 16.8 | 3714 | 111 | 26 | 1 | 0.28 | 0.01 | 32 |
| 18.5 | 3027 | 527 | 28 | 4 | 0.23 | 0.04 | 29 |
| 20.1 | 4140 | 105 | 38 | 4 | 0.32 | 0.01 | 21 |
| 20.9 | 3463 | 536 | 41 | 7 | 0.27 | 0.04 | 20 |
| 22.1 | 3453 | 455 | 35 | 4 | 0.26 | 0.03 | 23 |
| 23.3 | 3609 | 430 | 37 | 2 | 0.28 | 0.03 | 22 |
| *V* = (*V*_max_ × [O_2_]) × (*K*_m_ + [O_2_] × (1+[ O_2_]/*K*_s_))^-1^; *V* = respiration rate; *V*_max_ = maximum respiration rate; [O_2_] = substrate concentration (nmol O_2_ L^-1^); *K*_m_ = apparent half-saturation Michaelis-Menten constant; *K*_s_ = dissociation constant; *K*_s_ = 805.42 nmol O_2_ L^-1^; biological replicates = 3; oxic/anoxic shifts = 17; SE = standard errors. *R*_max_ = maximum respiration rate per cell | | | | | | | |
