# Supplementary material for: Microaerobic Lifestyle at Nanomolar O2 Concentrations Mediated by Low-Affinity Terminal Oxidases in Abundant Soil Bacteria
Source: mSystems. 2021 Jul 6;6(4):e00250-21. doi: 10.1128/mSystems.00250-21 (PMC8407424; doi:10.1128/mSystems.00250-21)
Supplement: TABLE S5 [file msystems.00250-21-st005.docx]

|  | | | | | | | |
| --- | --- | --- | --- | --- | --- | --- | --- |
| **Strain** | **Samples** | **Number of raw reads** | **Number of reads after error correction^a^** | **Number of reads w/o rRNA**^b^ | **% of rRNA**  **reads** | **Number of reads mapped to ORFs** | **% of mapped**  **reads to ORFs** |
| *Acidobacteriaceae* bacterium  KBS 83 | B-R1-10µM-t60 | 14931646 | 14863985 | 14841169 | 0.2% | 8840544 | 59.6% |
|  | B-R1-0.1µM-t60 | 13762630 | 13710578 | 13645544 | 0.5% | 6453810 | 47.3% |
|  | B-R1-0.001µM-t60 | 11266507 | 11235696 | 10923968 | 2.8% | 5432625 | 49.7% |
|  | B-R3-10µM-t60 | 13178238 | 13111866 | 13084736 | 0.2% | 7738466 | 59.1% |
|  | B-R3-0.1µM-t60 | 15008774 | 14966921 | 14870627 | 0.6% | 7390397 | 49.7% |
|  | B-R3-0.001µM-t60 | 12738897 | 12702002 | 12672231 | 0.2% | 5844715 | 46.1% |
|  | B-R4-10µM-t60 | 18196612 | 18114720 | 18086608 | 0.2% | 9630167 | 53.2% |
|  | B-R4-0.1µM-t60 | 14364618 | 14317715 | 14283864 | 0.2% | 7474979 | 52.3% |
|  | B-R4-0.001µM-t60 | 14094316 | 14048360 | 14019156 | 0.2% | 6649049 | 47.4% |
| *Terriglobus roseus* KBS 63 | K-R1-10µM-t60 | 16113250 | 16066848 | 13635505 | 15.1% | 9146503 | 67.1% |
|  | K-R1-0.1µM-t60 | 15895787 | 15875573 | 3121437 | 80.3% | 1910556 | 61.2% |
|  | K-R1-0.001µM-t10 | 8247403 | 8229140 | 6554200 | 20.4% | 3867186 | 59.0% |
|  | K-R1-0.001µM-t60 | 15860109 | 15838489 | 5568726 | 64.8% | 3462425 | 62.2% |
|  | K-R1-0µM-t15 | 16353224 | 16332688 | 5287554 | 67.6% | 3257520 | 61.6% |
|  | K-R2-10µM-t60 | 15378688 | 15363417 | 3182803 | 79.3% | 2062438 | 64.8% |
|  | K-R2-0.1µM-t60 | 14810287 | 14787406 | 8642432 | 41.6% | 4648377 | 53.8% |
|  | K-R2-0.001µM-t10 | 13885631 | 13874473 | 1242690 | 91.0% | 760977 | 61.2% |
|  | K-R2-0.001µM-t60 | 14372078 | 14360203 | 981522 | 93.2% | 562839 | 57.3% |
|  | K-R2-0µM-t15 | 14499752 | 14465272 | 9514579 | 34.2% | 5450405 | 57.3% |
|  | K-R3-10µM-t60 | 15293029 | 15270186 | 6313927 | 58.7% | 4038879 | 64.0% |
|  | K-R3-0.1µM-t60 | 14125016 | 14095094 | 9763387 | 30.7% | 5307470 | 54.4% |
|  | K-R3-0.001µM-t10 | 15480159 | 15462128 | 841909 | 94.6% | 467247 | 55.5% |
|  | K-R3-0.001µM-t60 | 14437742 | 14399475 | 13905824 | 3.4% | 7254241 | 52.2% |
|  | K-R3-0µM-t15 | 16938963 | 16924586 | 2627448 | 84.5% | 1414423 | 53.8% |
| *Acidobacterium capsulatum* 161 | A-R1-10µM-t60 | 16281952 | 16222470 | 16016862 | 1.3% | 11214363 | 70.0% |
|  | A-R1-0.1µM-t60 | 16566631 | 16505408 | 16491155 | 0.1% | 11415099 | 69.2% |
|  | A-R1-0.001µM-t10 | 12566560 | 12528947 | 12499767 | 0.2% | 8613115 | 68.9% |
|  | A-R1-0.001µM-t60 | 13677624 | 13635930 | 13378756 | 1.9% | 8694014 | 65.0% |
|  | A-R3-10µM-t60 | 14757807 | 14683340 | 14634225 | 0.3% | 9767691 | 66.7% |
|  | A-R3-0.1µM-t60 | 13434770 | 13389322 | 13372593 | 0.1% | 9380289 | 70.1% |
|  | A-R3-0.001µM-t10 | 14585380 | 14541544 | 14343829 | 1.4% | 9778275 | 68.2% |
|  | A-R3-0.001µM-t60 | 13885090 | 13843635 | 13648069 | 1.4% | 9088014 | 66.6% |
|  | A-R4-10µM-t60 | 16604510 | 16547909 | 16514524 | 0.2% | 11502196 | 69.6% |
|  | A-R4-0.1µM-t60 | 14228960 | 14186186 | 14169379 | 0.1% | 9862805 | 69.6% |
|  | A-R4-0.001µM-t10 | 15232953 | 15184107 | 15167639 | 0.1% | 10375043 | 68.4% |
|  | A-R4-0.001µM-t60 | 15173627 | 15114787 | 14909327 | 1.4% | 8811607 | 59.1% |
| ^a^RNA reads were quality-trimmed and error corrected using BBduk and Bayes-Hammer module of SPAdes assembler. ^b^rRNA reads were identified by mapping to the SILVA SSU132, LSU132, 5S rRNA databases. See text for details. B/K/A = one letter code for strains; R = replicate; µM = µmol O_2_ L^-1^; t60/t10/t15 = sampling timepoint after 60/10/15 minutes at the respective O_2_ concentration. | | | | | | | |
