# Supplementary material for: Microaerobic Lifestyle at Nanomolar O2 Concentrations Mediated by Low-Affinity Terminal Oxidases in Abundant Soil Bacteria
Source: mSystems. 2021 Jul 6;6(4):e00250-21. doi: 10.1128/mSystems.00250-21 (PMC8407424; doi:10.1128/mSystems.00250-21)
Supplement: TABLE S3 [file msystems.00250-21-st003.docx]

| **Oxygen  (µmol L^-1^)** | **Time^a^ (min)**  **at [O_2_]** | **Sampling**  **timepoint** |
| --- | --- | --- |
| 10 | 60 | 10-t60 |
| 1 | 0 | 1-t0 |
|  | 10 | 1-t10 |
|  | 60 | 1-t60 |
| 0.1 | 0 | 0.1-t0 |
|  | 10 | 0.1-t10 |
|  | 60 | 0.1-t60 |
| 0.001^b^ | 0 | 0.001-t0 |
|  | 10 | 0.001-t10 |
|  | 60 | 0.001-t60 |
| 0^c^ | 15 | 0-t15 |
| ^a^Time is relative to stable O_2_-concentration readings: t0 = sampling as immediate as the LUMOS reading indicated a stable signal after the switch to the new O_2_ concentration (equilibrium of O_2_ consumption by cells and N_2_-air gas inflow), t10 = sampling after reading was stable for 10 min, t60 = sampling after reading was stable for 60 min, t15 = sampling after 15 min of anoxia while cultures were only purged with N_2_ gas.  ^b^0.001 µmol O_2_ L^-1^ is close to the detection limit of LUMOS and represents an “apparent anoxia” as O_2_ was still provided but not resolved as too close to the detection limit.  ^c^Detection limit of LUMOS = 0.0005 µmol O_2_ L^-1^, hence 0 µmol O_2_ L^-1^ is < 0.0005 µmol O_2_ L^-1^. | | |
