# Supplementary material for: Microaerobic Lifestyle at Nanomolar O2 Concentrations Mediated by Low-Affinity Terminal Oxidases in Abundant Soil Bacteria
Source: mSystems. 2021 Jul 6;6(4):e00250-21. doi: 10.1128/mSystems.00250-21 (PMC8407424; doi:10.1128/mSystems.00250-21)
Supplement: TABLE S4 [file msystems.00250-21-st004.docx]

|  | | | | | | | | |
| --- | --- | --- | --- | --- | --- | --- | --- | --- |
| **Species name** | **Target protein** | **HCO^1^ (sub)family** | **Target**  **gene** | **IMG locus tag of target gene** | **Primer name** | **Primer sequence (5'-3')** | **Product**  **size (bp)** | **PCR efficiency** |
| ***Acidobacteriaceae* bacterium**  **KBS 83** | *caa*_3_-type cytochrome c oxidase subunit I | A1 | *ctaD* | G002DRAFT_03882 | A1B2-F A1B2-R | CTC ATC GCC AAT TTC AGC CG GTG GAG GTA CTG ATC GAG CG | 333 | 1.86 |
|  | *caa*_3_-type cytochrome c oxidase subunit I | A1 | *ctaD* | G002DRAFT_03417 | A2B2-F A2B2-R | TCT GAT TGT CTC CGG CTT CG GGC AGC ACC AGT ACG TAG AC | 300 | 1.87 |
|  | *caa*_3_-type cytochrome c oxidase subunit I | A1 | *ctaD* | G002DRAFT_04166 | A3B2-F A3B2-R | GTT TGC CTG GCT CAT GTT CG CTG ACA AAG GCA ATA GCG GC | 300 | 1.79 |
|  | *caa*_3_-type cytochrome c oxidase subunit I | A2 | *ctaD* | G002DRAFT_04897 | A4B-F A4B-R | CCA CGG CAT CAT CAT GGT CT TTC AGC CCG GTA AAG ATG GAG | 300 | 1.77 |
|  | DNA-directed RNA polymerase subunit β | N/A^2^ | *rpoB* | G002DRAFT_00011 | rpoB2-F rpoB2-R | GCC ATC CGC GAG TTC TTC GCG ACT CGA TGA AGC CGT AC | 268 | 1.88 |
| ***Terriglobus roseus* KBS 63** | *caa*_3_-type cytochrome c oxidase subunit I | A2 | *ctaD* | Terro_0292 | A1K-F A1K-R | TCT CCC TGC TGA CAA TGC TG ACA TAC GGC CGG TCA TCT TG | 300 | 1.95 |
|  | *caa*_3_-type cytochrome c oxidase subunit I | A2 | *ctaD* | Terro_4044 | A2K-F A2K-R | CAC GAT TGT TCT GGT TGC GG AAG ACC AGC GCT GAG TAC TG | 300 | 2.02 |
|  | *cbb*_3_-type cytochrome c oxidase subunit I | C | *ccoN* | Terro_4248 | CK-F CK-R | ACT CGT CAT CAT CGC CAT CC TAC GAT CCA CCA GCA GAG GA | 283 | 1.96 |
|  | DNA-directed RNA polymerase subunit β | N/A^2^ | *rpoB* | Terro_3686 | rpoB2-F rpoB2-R | GCC ATC CGC GAG TTC TTC GCG ACT CGA TGA AGC CGT AC | 268 | 1.94 |
| ***Edaphobacter* sp. TAA 166** | *caa*_3_-type cytochrome c oxidase subunit I | A2 | *ctaD* | H979_RS0112685 | A1T1-F A1T1-R | CTC TAC GCC TTC GCC TTC AT ACG AGG CAT TCC CAG GTA AC | 300 | 1.90 |
|  | *caa*_3_-type cytochrome c oxidase subunit I | A1 | *ctaD* | H979_RS0124555 | A2T1-F A2T1-R | GAT GGT CTT CTT CGT GGG CA GTC GTA ACG ATG TTC AGC GC | 327 | 1.93 |
|  | cytochrome *bd*-type quinol oxidase | N/A^2^ | *cydA* | H979_RS0125960 | BDT1-F BDT1-R | ACT TGG TCT GCT ATG GCC AC GCT GCG AAC GGA TGA ATG TC | 285 | 1.92 |
|  | DNA-directed RNA polymerase subunit β | N/A^2^ | *rpoB* | H979_RS0102725 | rpoB2-F rpoB2-R | GCC ATC CGC GAG TTC TTC GCG ACT CGA TGA AGC CGT AC | 268 | 2.02 |
| ***Acidobacterium capsulatum* 161** | *caa*_3_-type cytochrome c oxidase subunit I | A1 | *ctaD* | ACP_2437 | AA-F AA-R | TGA TTG ACC GCT ACC TTG GC GCA TGG TGG AAA GCA CGA AG | 300 | 1.95 |
|  | *cbb*_3_-type cytochrome c oxidase subunit I | C | *ccoN* | ACP_0885 | CA3-F CA3-R | GTC TGA AGG CCG GTA AAC CA AAA TCC TGC CAT GGC CAT GA | 338 | 1.88 |
|  | cytochrome *bd*-type quinol oxidase | N/A^2^ | *cydA* | ACP_0468 | BDA3-F BDA3-R | GCC TCT TCC ACT CGG AGA AG GCA CCC AAC GCG ATT GAT AC | 300 | 1.94 |
|  | DNA-directed RNA polymerase subunit β | N/A^2^ | *rpoB* | ACP_2928 | rpoB2-F rpoB2-R | GCC ATC CGC GAG TTC TTC GCG ACT CGA TGA AGC CGT AC | 268 | 1.97 |
| ^1^HCO = heme-copper oxidase, ^2^N/A = not applicable | |  |  |  |  |  |  |  |
